# Stroma-Rich Co-Culture Multicellular Tumor Spheroids as a Tool for Photoactive Drugs Screening

**DOI:** 10.3390/jcm8101686

**Published:** 2019-10-15

**Authors:** Ilya Yakavets, Samuel Jenard, Aurelie Francois, Yulia Maklygina, Victor Loschenov, Henri-Pierre Lassalle, Gilles Dolivet, Lina Bezdetnaya

**Affiliations:** 1 Centre de Recherche en Automatique de Nancy, Centre National de la Recherche Scientifique UMR 7039, Université de Lorraine, Campus Sciences, Boulevard des Aiguillette, 54506 Vandoeuvre-lès-Nancy, France; i.yakavets@nancy.unicancer.fr (I.Y.);; 2 Research Department, Institut de Cancérologie de Lorraine, 6 avenue de Bourgogne, 54519 Vandoeuvre-lès-Nancy, France; 3 Laboratory of Biophysics and Biotechnology, Belarusian State University, 4 Nezavisimosti Avenue, 220030 Minsk, Belarus; 4 Institut Jules Bordet, Surgery Department, Université Libre de Bruxelles, Boulevard de Waterloo 121, 1000 Brussel, Belgium; 5 Prokhorov General Physics Institute of the Russian Academy of Sciences, Vavilova 38, 117942 Moscow, Russia; 6 National Research Nuclear University MEPhI, Kashirskoe shosse, 31, 115409 Moscow, Russia

**Keywords:** photodynamic therapy, temoporfin, head and neck squamous carcinoma, multicellular tumor spheroids, cancer-associated fibroblasts, drug penetration

## Abstract

Conventional 3D multicellular tumor spheroids of head and neck squamous cell carcinoma (HNSCC) consisting exclusively of cancer cells have some limitations. They are compact cell aggregates that do not interact with their extracellular milieu, thus suffering from both insufficient extracellular matrix (ECM) deposition and absence of different types of stromal cells. In order to better mimic in vivo HNSCC tumor microenvironment, we have constructed a 3D stroma-rich in vitro model of HNSCC, using cancer-associated MeWo skin fibroblasts and FaDu pharynx squamous cell carcinoma. The expression of stromal components in heterospheroids was confirmed by immunochemical staining. The generated co-culture FaDu/MeWo spheroids were applied to study penetration, distribution and antitumor efficacy of photoactive drugs such as Temoporfin and Chlorin e6 used in the photodynamic therapy flow cytometry and fluorescence microscopy techniques. We also investigated the distribution of photodiagnostic agent Indocyanine Green. We demonstrated that the presence of stroma influences the behavior of photoactive drugs in different ways: (i) No effect on Indocyanine Green distribution; (ii) lower accumulation of Chlorin e6; (iii) better penetration and PDT efficiency of Temoporfin. Overall, the developed stroma-rich spheroids enlarge the arsenal of in vitro pre-clinical models for high-throughput screening of anti-cancer drugs.

## 1. Introduction

Improvement in cancer drug development success rates is strictly related to the use of physiologically relevant in vitro cellular models [1]. These models should recapitulate the morphology, microenvironment, cell-cell and cell-stroma interactions inherent to solid tumors. The conventional 2D flat tumor cell cultures are too far from in vivo situation, lacking three-dimensionality, heterogeneity, extracellular matrix and penetration barriers, all these existing in solid tumors in vivo. Thus, translating of therapeutics into clinical reality is a real hurdle. To fulfill the gap between monolayer cells and in vivo xenografted animals, more sophisticated in vitro cell models have been developed (i.e., multilayers, spheroids, microtissues) [2]. There is a broad consensus that 3D tumor culture models considerably improve the predictive potential of the in vivo efficacy of various anticancer drugs [3]. Multicellular tumor spheroids (MCTS) are the most widely used 3D in vitro model in preclinical cancer research offering cell-cell and cell-matrix interactions that mimic the native tumor environment [4]. To establish 3D models, most studies are focusing on a single cell line. However, such homospheroids consisting exclusively of cancer cells have some limitations. They are compact cell aggregates that do not interact with their extracellular milieu, thus suffering from insufficient extracellular matrix (ECM) deposition and absence of different types of stromal cells. The stromal interactions exhibited in 3D tumor models can influence drug penetration, therapeutic response, tumor progression and multicellular resistance. Therefore, in order to achieve a more realistic in vivo microenvironment, not only tumor cells but also stromal cells (e.g., fibroblasts, immune cells…) should be considered. 

Head and neck squamous cell carcinoma (HNSCC) is the sixth most common malignant tumor in the world with more than 300,000 deaths each year [5]. To date, a certain number of studies demonstrated that HNSCC spheroids [6,7,8,9,10,11,12,13,14,15,16,17] are a promising model reproducing immunochemical aspects and characteristics of tumor tissue which could be applied for high throughput screening of anti-cancer theranostics [1,18]. It is worth noting that these studies used spheroids composed of a single cell line, while immunohistochemical analysis showed that 60–70% of HNSCC tumors are rich in cancer-associated fibroblasts (CAFs) [19,20]. CAFs are the primary source of ECM components providing significant physical resistance for effective drug delivery [21]. Thus, a more relevant 3D HNSCC spheroid model is required for screening of molecular and nanoformulated drugs.

The present study was aimed at the development of 3D co-culture stroma-rich MCTS consisting of HNSCC cancer and stromal (CAF) cells in order to better mimic in vivo tumor microenvironment. The generated stroma-rich co-culture spheroids were applied to study the penetration, diffusion and antitumor efficacy of photoactive drugs used in Photodiagnosis (PD) and Photodynamic therapy (PDT) of cancers. To date, photoactive compounds are widely used in the management of HNSCCs. PDT, which is based on the interaction between a photosensitizer (PS), appropriate wavelength and oxygen to cause cell death [22,23], was already reported as an effective treatment modality for oral malignant disorders and HNSCC [24,25]. Among clinically relevant PSs we were interested in Temoporfin (mTHPC) and Chlorin e6 (Ce6). Temoporfin is clinically approved in the European Union in 2001 for the treatment of HNSCC [26,27], while Ce6 demonstrated to be effective in the management of pre-malignant and malignant head and neck disorders [25]. Further, near infra-red (NIR) fluorescent tracers such as Indocyanine Green (ICG) attracts attention in PDT and especially in PD due to the deep infrared light penetration across the tissues. ICG, one of the most common dyes used in NIR fluorescent image-guided surgery [28,29] was applied for sentinel node mapping and was considered effective for intraoperative imaging of head and neck lesions [30].

## 2. Experimental Section

### 2.1. Chemical and Reagents

mTHPC was kindly provided by biolitec research GmbH (Jena, Germany). mTHPC stock solution (1 mM) was prepared in absolute ethanol (99.6%). The chlorin e6 (Ce6, Frontier Scientific, Logan, UT, USA) stock solution was prepared at 1.5 mM in dimethylsulfoxide (DMSO, ThermoFisher, Waltham, MA, USA). Indocyanine green (ICG) powder (Infracyanine, SERB, Paris, France) was extemporaneously diluted at 1.5 mM in phosphate buffer saline (PBS, GIBCO™, ThermoFisher, Waltham, MA, USA).

### 2.2. Cell Culture

The FaDu (human pharynx squamous cell carcinoma) cell line was purchased from ATCC (Cat. No: ATCC1 HTB-43™). Cells were cultured in phenol red-free Roswell Park Memorial Institute 1640 medium (RPMI-1640, Invitrogen™, Carlsbad, California, USA), supplemented with 9% (*v*/*v*) heat-inactivated fetal bovine serum (FBS, Sigma-Aldrich, St. Louis, MO, USA), penicillin (10,000 IU) streptomycin (10,000 mg/mL) and 1% (*v*/*v*) 0.2 M glutamine (Invitrogen™, Carlsbad, California, USA). MeWo cells (ATCC HTB-65^TM^), granular fibroblasts, derived from human melanoma [31], were used as CAF. Cells were cultured in Minimal Essential Medium (MEM, Sigma-Aldrich, St. Louis, MO, USA) supplemented with 9% (*v*/*v*) of FBS and 1 mM sodium pyruvate. Cells were kept as a monolayer culture in a humidified incubator (5% CO_2_) at 37 °C. Cell culture was reseeded every week to ensure exponential growth.

### 2.3. Spheroids Formation

MCTS were generated from FaDu cells using the liquid overlay technique (LOT), as described previously [32]. Briefly, 100 µL of FaDu cells (5 × 10^4^ cells/mL) and 100 µL of full RPMI medium were added to each well of a 96-well plate previously coated with 1% agarose (*w*/*v* in water) and cultured at 37 °C, 5% CO_2_ for 5 days before being taken into experiments. Co-culture spheroids were constructed by seeding FaDu cells (100 µL at 5 × 10^4^ cells/mL) simultaneously with 100 µL of MeWo cells in various concentrations, from 0.5 to 10 × 10^4^ cells/mL. The morphology and size of spheroids were monitored from day 3 after seeding until day 10 by bright field microscopy using an inverted Olympus CK2 microscope (Olympus, Rungis, France). From 8 to 16 spheroids were used for each experimental condition. At days 3, 5, 7 or 10 after seeding, spheroids were embedded into resin Shandon^TM^ Cryomatrix^TM^ (ThermoFisher, Waltham, MA, USA), frozen, cut and 10 µm thick sections were further used for fluorescence microscopy and immunohistochemistry analysis.

### 2.4. Fluorescence Staining

To distinguish two types of cells in spheroid co-culture, MeWo cells were pre-stained with a membrane green fluorescent cell marker PKH67 (Sigma-Aldrich, St. Louis, MO, USA) before seeding with FaDu cells. The pre-staining of MeWo cells was performed following the manufacturer instructions. Briefly, the suspension of 10^7^ MeWo cells was washed once with serum-free medium. The cell pellet was then gently mixed in the dark with 4 µM of PKH67 in the solution provided by manufacturer for 10 min. The labeling was stopped with the addition of two volumes of fetal bovine serum for 2 min and then washed twice in complete medium before co-seeding with FaDu cells into agarose pre-coated plates. The efficiency and stability of membrane staining were checked by flow cytometry in MeWo cells immediately after staining and in co-cultured MCTS 5 days after seeding. 

Before incubation with drugs (mTHPC, Ce6, and ICG), spheroids were washed with serum-free RPMI medium. 100 µL of complete medium was carefully removed from the plates and 100 µL of twice concentrated drug solution, prepared in medium supplemented with 2% of serum, was added to MCTSs for the final drug concentration of 4.5 μM. Cells were kept in a humidified incubator (5% CO_2_) in the dark at 37 °C. At appropriate times, after washing with PBS, MCTSs were embedded into the resin matrix and 10 µm thick sections were used for fluorescence microscopy. For further analysis we used the cryosections with the diameter of spheroid section about 450 μm corresponding to the central part of spheroid.

### 2.5. Analytical Techniques

#### 2.5.1. Fluorescence Microscopy 

Fluorescence images were collected from both intact spheroids and spheroids cryo-sections. Intact spheroids were washed in serum-free RPMI medium and directly placed at the slides. Fluorescence was observed under an upright epifluorescence microscope (AX-70 Provis, Olympus, Paris, France). PKH67 fluorescence was observed using 460–490 nm excitation bandpass filter associated with a 505 nm dichroic mirror and 510–550 nm emission bandpass filter. The fluorescence images of mTHPC, Ce6 and ICG were obtained using the filter set at 405–445 nm excitation associated with a 570 nm dichroic mirror and a 590 nm long-pass emission filter for fluorescence measurements. The observation of FITC-Annexin V stained cells was performed using an excitation filter 460–490 nm and an emission filter with a bandpass of 510–550 nm. Fluorescence images of the whole spheroid were recorded using 4× objective. 

The analysis of images was performed with ImageJ (NIH, Bethesda, MD, USA) software. To estimate the dye penetration profile in spheroid, the special macros was proposed [33]. Briefly, the spheroid area was divided into 100 concentric rims with a linearly decreasing diameter. After that, the mean intensity of pixels in each rim was calculated. The final profiles were plotted as mean ± standard deviation from different cryo-sections (*n* = 4–9).

#### 2.5.2. Histology and Immunochemistry Analysis

The frozen sections were fixed in 4% formaldehyde solution for 1 min and rinsed with water before staining for histology study and further immunohistochemical characterization. 

Structural characterization of MCTS was performed with HES (Hematoxylin-Eosin-Safran) automated device (Dako CoverStainer, Dako, Santa Clara, CA, USA). The extracellular matrix was evidenced by different markers such as vimentin, alpha-smooth muscle actin (α-SMA), fibronectin, and collagen. Vimentin and proliferative marker Ki-67 staining were performed with Benchmark Ultra Automate (Ventana, Tucson, AZ, USA). Vimentin antibody was diluted at 1:200 (Clone V9; Dako, Santa Clara, CA, USA); the dilution of Ki67 antibody (clone Mib1; Dako, Santa Clara, CA, USA) was 1:50. 

For staining with fibronectin and α-SMA, sections were fixed with 4% paraformaldehyde solution and rinsed with water. Endogenous peroxidase activity was blocked in a 3% hydrogen peroxide solution for 5 min and afterward washed with 0.1% Tween in PBS (PBST). Primary antibodies (Abcam, Cambridge, UK) were diluted 1:100 and 1:50 for α-SMA and fibronectin respectively and incubated overnight at 4 °C in 1% bovine serum albumin solution. After 3 washings in PBST, sections were stained for 1 h with the biotinylated secondary antibody (diluted as 1:200) and then incubated in streptavidin-peroxidase for 30 min at the room temperature in the dark. After washings, bound peroxidase was identified using the NovaRED system (Vector laboratories, Burlingame, CA, USA) and nuclear counterstaining with hematoxylin coloration was performed. 

Collagen fibers in MCTS were stained with Picro Sirius Red stain kit (Abcam, Cambridge, UK) according to the manufacturer instructions. Briefly, sections were covered with Picro Sirius Red solution for 1 h at room temperature and then rapidly rinsed twice with an acetic acid solution. After that, the slides were washed with absolute alcohol, dehydrated and mounted for light microscopy analysis, where the collagen positive regions appear in red, while cytoplasm remained yellow. 

#### 2.5.3. Flow Cytometry

In order to dissociate MCTSs, they were transferred into a 12-well plate, washed twice with PBS, incubated with 0.025% trypsin (GIBCO™, ThermoFisher, Waltham, MA, USA) and 0.01% ethylenediaminetetraacetic acid (EDTA, GIBCO™, ThermoFisher, Waltham, MA, USA). Afterwards, the plate with spheroids was protected from light, placed on the rotatory shaker (60 rpm) for 20–25 min and then 3 mL of the complete culture medium was added to inhibit trypsinization. Finally, spheroids were resuspended, centrifuged (1500 rpm, 5 min) and the pellet was resuspended in the fresh culture medium.

Flow cytometry analysis was performed using FACSCalibur (BD, Franklin Lakes, NJ, USA), equipped with lasers emitting at 488 nm and 633 nm. The fluorescence of PKH67 was detected in the fluorescence channel FL2 with a 585 ± 42 nm filter under the excitation at 488 nm, while the detection of photoactive drugs (mTHPC, Ce6, and ICG) was performed in FL4 channel with 661 ± 16 nm filter under the excitation at 633 nm. Propidium iodide (PI) fluorescence was detected in FL3 channel with a 670 nm longpass filter (excitation at 488 nm). Data analysis was carried out using Flowing Software (Turku Centre for Biotechnology, Turku, Finland).

#### 2.5.4. Photoirradiation

For photo-toxicity experiments, spheroids were incubated for 24 h with mTHPC or Ce6 at 37 °C, transferred to the agarose-coated 35 mm Petri dishes and then subjected to irradiation. Irradiation was performed at 652 nm with a Ceralas PDT diode laser (CeramOptec GmbH, Bonn, Germany) at 20 J/cm^2^ (fluence rate of 30 mW/cm^2^). Control spheroids were exposed to the drug only (drug, no light). 

To estimate the photo-induced damage, we used two fluorescence-based techniques: fluorescence microscopy imaging of apoptotic and necrotic cells and flow cytometry analysis of necrotic cells. Intact homo- and hetero-spheroids, taken 5 h post-PDT, were stained for 1 h with 4.5 µg/mL FITC-Annexin V (Biolegend, San Diego, CA, USA) for imaging. For flow cytometry analysis, MCTSs were dissociated 6 h post-PDT, and the obtained cell suspension was stained with 1 µg/mL PI (Biolegend, San Diego, CA, USA) for 15 min at room temperature.

### 2.6. Statistics

The data from at least three independent experiments are presented as mean ± standard deviation. The data were evaluated using nonparametric Mann Whitney’s *U* test with a significant level of *p* < 0.05.

## 3. Results

### 3.1. Formation and Characterization of Multicellular Tumor Spheroids

#### 3.1.1. Growth Kinetics

**Figure 1:**
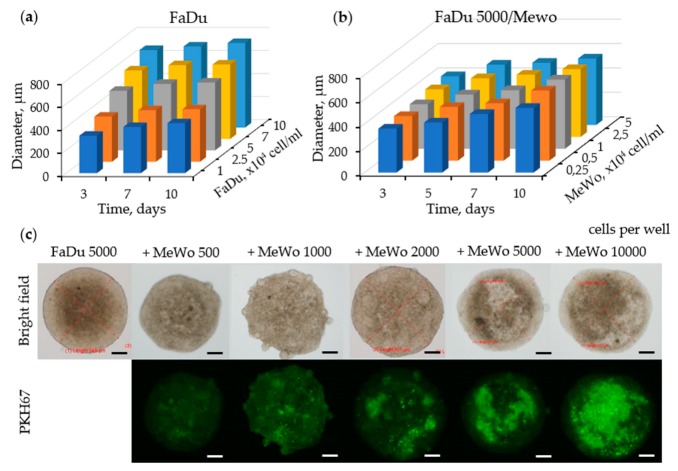
Spheroids characterization. Growth kinetics of (**a**) monoculture FaDu and (**b**) co-culture FaDu/MeWo multicellular spheroids. (**c**) Representative optical and fluorescence imaging of co-culture FaDu/MeWo multicellular spheroids captured on day 5 after seeding. Scale bars: 100 µm.

The formation of FaDu homospheroids was performed by seeding cancer cells in agarose-coated 96-well plates at different densities: 1; 2.5; 5; 7; 10 × 10^4^ cells/mL corresponding to 2000; 5000; 10,000; 14,000 and 20,000 cells per well respectively. The kinetics of spheroids growth is demonstrated in Figure 1a. Initial spheroids size is tightly related to initial cell concentration. For each initial concentration, FaDu spheroids were growing slowly and an increase in the diameter never exceeded 10% at the end of incubation. For further experiments, we have selected the seeding concentration 2.5 × 10^4^ cells/mL (5000 cells per well) providing spheroids with 500 µm in diameter. 

Co-culture spheroids mimicking tumor-stroma interactions were constructed using MeWo cancer-associated fibroblasts, derived from human melanoma metastatic sites [31]. The addition of MeWo cells at the concentration of 0.25–5 × 10^4^ cells/mL (500–10,000 cells per well) to FaDu (2.5 × 10^4^ cells/mL) cells only slightly changed the size of the resulting spheroids compared to homospheroids (Figure 1b). If the average size of FaDu homospheroids 5 days after culturing was 427 ± 18 μm, the size of co-cultured FaDu/MeWo spheroids was 429 ± 18 μm, 439 ± 16 μm, 443 ± 22 μm, 478 ± 22 μm and 496 ± 28 μm at MeWo cells seeding concentrations of 0.25; 0.5; 1; 2.5 and 5 × 10^4^ cells/mL, respectively.

We investigated the arrangement of tumor cells and fibroblasts into spheroids during the culture period. The bright field and fluorescence images of intact spheroids at different FaDu:MeWo seeding concentration ratios are presented in Figure 1c. MeWo cells were stained with PKH67 green dye before seeding in order to distinguish the distribution of fibroblasts in FaDu/MeWo heterospheroids. Optical imaging at 5 days post-seeding revealed the formation of reproducible sphere-shaped 3D homo- and heterospheroids. Co-culture spheroids FaDu/MeWo at 5:2 ratio (F5M2, 5000 FaDu cells and 2000 MeWo cells per well) and 5:5 ratio (F5M5, 5000 FaDu cells and 5000 MeWo cells per well) were selected for further experiments.

**Figure 2:**
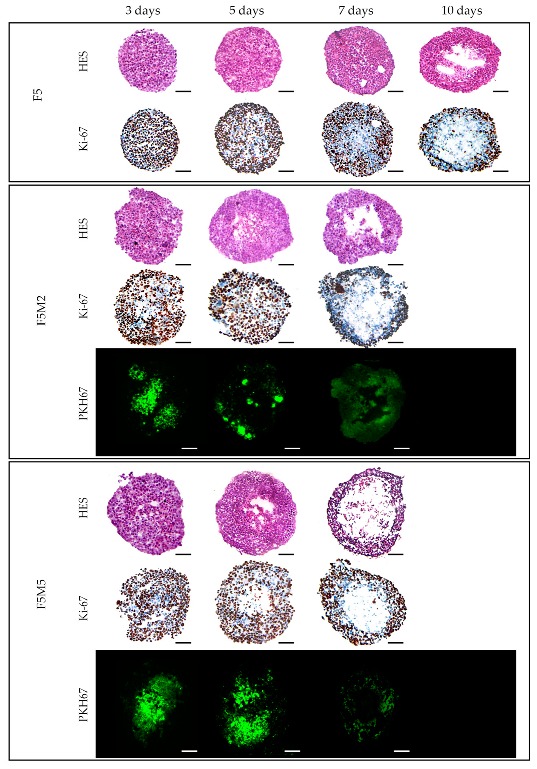
Typical cryosections images of FaDu monoculture (F5) and FaDu:MeWo (5:2 and 5:5) co-culture spheroids stained with HES and Ki-67 at days 3, 7 and 10 days post-seeding. MeWo cells were additionally pre-stained with PKH67 green fluorescent dye. Scale bars: 100 µm.

For in-detail characterization of the spatial and temporal distribution of cells, spheroids at days 3, 5 and 7 post-seeding were isolated, frozen-cut and cryosections were subsequently stained with HES and Ki-67 (Figure 2). On day 3, HES staining revealed the uniform cell distribution and a compact core with tightly adhered cells in both homo-and heterospheroids. The necrotic zone was strongly expressed on day 10 in FaDu spheroids, while FaDu:MeWo 5:2 co-culture spheroids demonstrated necrotic area from already day 7. The subsequent enrichment of FaDu spheroids with fibroblasts (FaDu:MeWo 5:5) resulted in the formation of necrotic core on day 5. These results were also confirmed by Ki-67 staining. Considering above studied parameters (cell distribution patterns, necrotic area, proliferative capacity), we have selected day 5 post-seeding for further experiments.

#### 3.1.2. Expression of Stroma Markers

**Figure 3:**
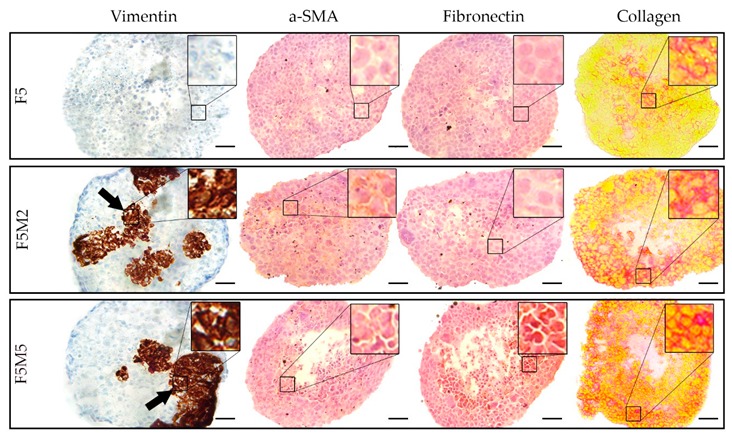
Microscopic images of cryosections of homo- (F5) and heterospheroids (F5M2 and F5M5) at day 5 post-seeding, immunohistologically stained with vimentin, α-SMA, fibronectin and collagen markers. The enlarged (3.5×) regions of interest (black squares) are presented in the upper right of each image. Scale bars: 50 µm.

The fibroblasts distribution patterns in co-cultured spheroids was established at both FaDu/MeWo ratios and at all post-seeding times (Figure 2). An increase in fibroblasts content (FaDu:MeWo 5:5) resulted in their localization mostly in the center of the spheroid forming the clusters. In order to characterize the expression of stromal biomarkers in co-culture spheroids, specific fluorescence immunostaining was performed on the cryosections at day 5 post-seeding (Figure 3). Compared to homospheroids, co-culture spheroids were positively stained for activated fibroblasts markers as vimentin and α-SMA [34,35,36]. Vimentin staining demonstrated that fibroblasts tend to form large clusters and their size increases with an increase in fibroblasts concentration. Finally, the induction of ECM components in 3D tissue model was confirmed by ECM biomarkers such as fibronectin and collagen in FaDu:MeWo heterospheroids compared to FaDu homospheroids. Indeed, FaDu:MeWo 5:2 and FaDu:MeWo 5:5 heterospheroids display the presence of ECM collagen across the whole volume of spheroids.

### 3.2. Screening of Photoactive Drugs

The developed stroma-rich FaDu:MeWo co-culture spheroids were used to study the behavior of fluorescent drugs applied for PD and PDT. With this aim, we selected two PSs widely used in PDT as mTHPC and Ce6 and clinically approved PD agent ICG. All these fluorescent dyes were tested for their accumulation and distribution in the developed 3D tumor models. In addition, mTHPC- and Ce6-mediated PDT efficiency was estimated and compared in various spheroid models.

#### 3.2.1. Fluorescence Distribution Patterns 

**Figure 4:**
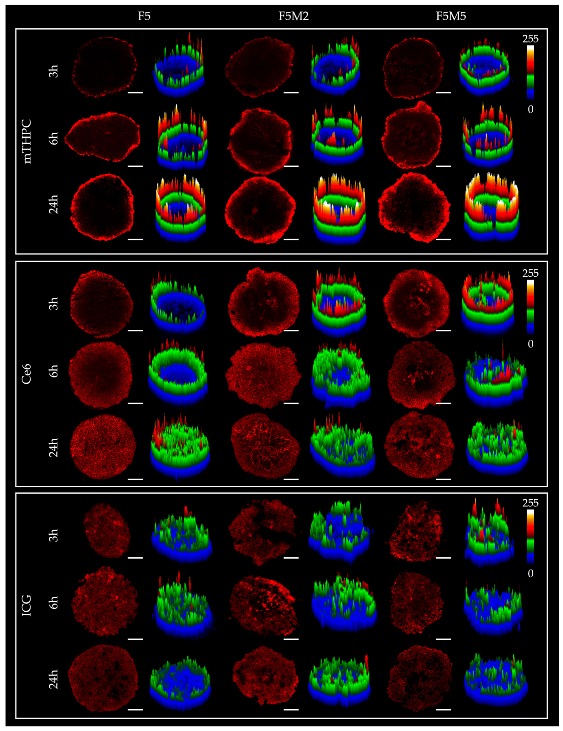
The typical fluorescence images of cryosections of FaDu monoculture (F5) and FaDu:MeWo (5:2 and 5:5) spheroids at 5 day post-seeding after incubation with mTHPC, Ce6 (4.5 µM) and ICG (40 µM) for 3, 6 and 24 h. mTHPC fluorescence is displayed in red-color (2D images) and pseudo-colors (3D surface plots).

**Figure 5:**
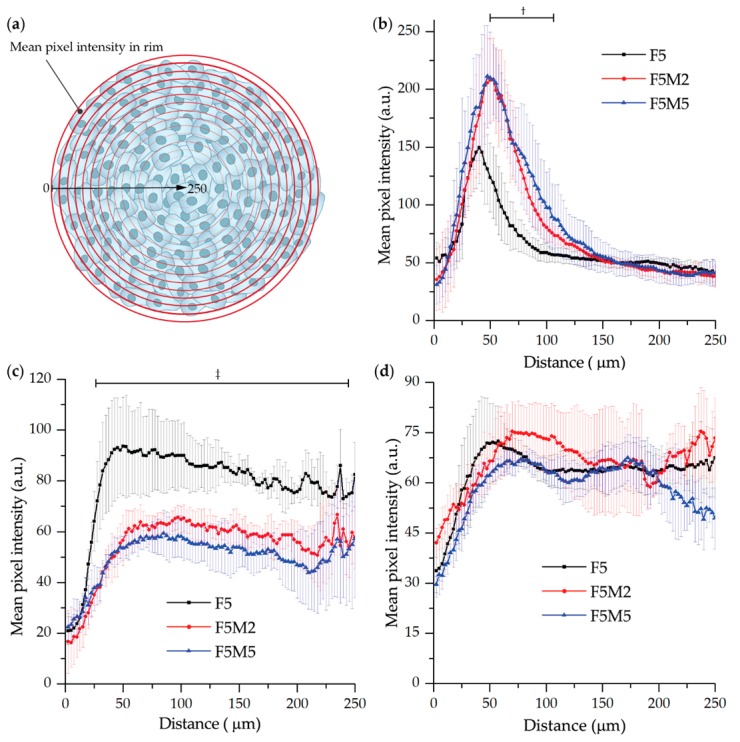
(**a**) The scheme of estimation of penetration profile in spheroid cryosections. (**b**–**d**) Penetration profiles of (**b**) mTHPC, (**c**) Ce6 (4.5 µM) and (**d**) ICG (40 µM) in FaDu (black), FaDu:MeWo 5:2 (red) and FaDu:MeWo 5:5 (blue) spheroids at 5-day post-seeding after incubation for 24 h. ^†^: statistically significant (*p* < 0.05) difference in the range of 50–110 µm between F5 and F5M2/F5M5 treated with mTHPC, ^‡^: statistically significant (*p* < 0.05) difference in the range of 20–240 µm between F5 and F5M2/F5M5 treated with Ce6.

The influence of stroma components on the penetration of molecular dyes in 3D tumor tissue models was studied by fluorescence microscopy. Spheroids were incubated 3, 6 and 24 h with mTHPC, Ce6 and ICG, frozen-cut with following cryosections analysis (Figure 4). For a better comparison of the dye distribution in spheroids, the surface plots of fluorescence patterns were added. We also estimated the penetration profiles of dyes in spheroids calculating the mean fluorescence intensity of pixels in each equidistant rim at 24 h incubation (Figure 5). 

As seen in Figure 4, mTHPC is confined to the periphery of FaDu spheroids irrespective of incubation time, with the higher fluorescence intensity at longer incubation times. The addition of fibroblasts increases the overall mTHPC fluorescence in both co-cultured spheroid models (Figure 5b). Moreover, mTHPC penetrates slightly deeper in stroma-rich MCTSs compared to FaDu homospheroids (statistically significant difference in the range of 50–100 µm of penetration distance, *p* < 0.05). In contrast, Ce6 completely penetrates into monoculture FaDu spheroids and this process takes about 24 h, while in co-culture FaDu:MeWo (F5M2 and F5M5) spheroids almost homogeneous distribution of Ce6 was observed at already 6 h incubation (Figure 4). However, while Ce6 is homogeneously distributed in all spheroid models (24 h), co-culture spheroids exhibited lower overall fluorescence than FaDu homospheroids (statistically significant difference in the range of 20–240 µm of penetration distance, *p* < 0.05) (Figure 5). As for ICG, it rapidly penetrates in-depth of FaDu spheroids irrespective of the presence of ECM, demonstrating homogeneous distribution in spheroids at all incubation times.

#### 3.2.2. Distribution of Drugs in Homo- and Heterospheroids

**Figure 6:**
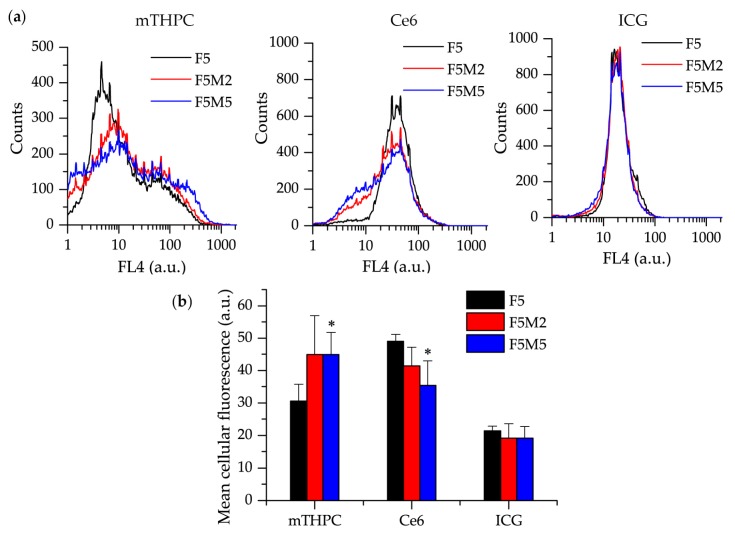
(**a**) The comparison between the distribution profiles of mTHPC, Ce6 (4.5 µM), and ICG (40 µM) in FaDu (black), FaDu:MeWo 5:2 (red) and FaDu:MeWo 5:5 (blue) spheroids after 24 h incubation. (**b**) The mean fluorescence intensity of the drugs in F5, F5M2 and F5M5 spheroid cells, after 24 h incubation with mTHPC (4.5 µM), Ce6 (4.5 µM) and ICG (40 µM). *: statistically significant (*p* < 0.05) difference compared to FaDu homospheroids samples.

In order to introduce the quantitative aspect in the behavior of fluorescent dyes in stroma-rich FaDu:MeWo spheroids, we performed a flow cytometry analysis. Spheroids were incubated 24 h with dyes, trypsinized and cell suspension was analyzed by flow cytometry (Figure 6). Broad distribution profile with several peaks was observed in mTHPC-treated FaDu spheroids (Figure 6a). In co-culture FaDu:MeWo spheroids, the distribution profile was broader with the increased fraction of highly fluorescent cells thus indicating a significant increase of total mTHPC content in heterospheroids (Figure 6b). Contrary to mTHPC, the profile of Ce6 distribution in FaDu spheroids was characterized by a narrow peak pointing out a homogeneous distribution across spheroid (Figure 6a). In FaDu:MeWo heterospheroids, we observed the presence of the additional fraction of cells with low Ce6 fluorescence. Hence, in contrast to mTHPC, the total concentration of Ce6 in FaDu:MeWo was lower compared with FaDu homospheroids (Figure 6b). A completely different situation was observed for ICG distribution profile in spheroids. The latter were identical for both homo- or heterospheroids (Figure 6a,b), thus ruling out the influence of stroma components on ICG distribution.

**Figure 7:**
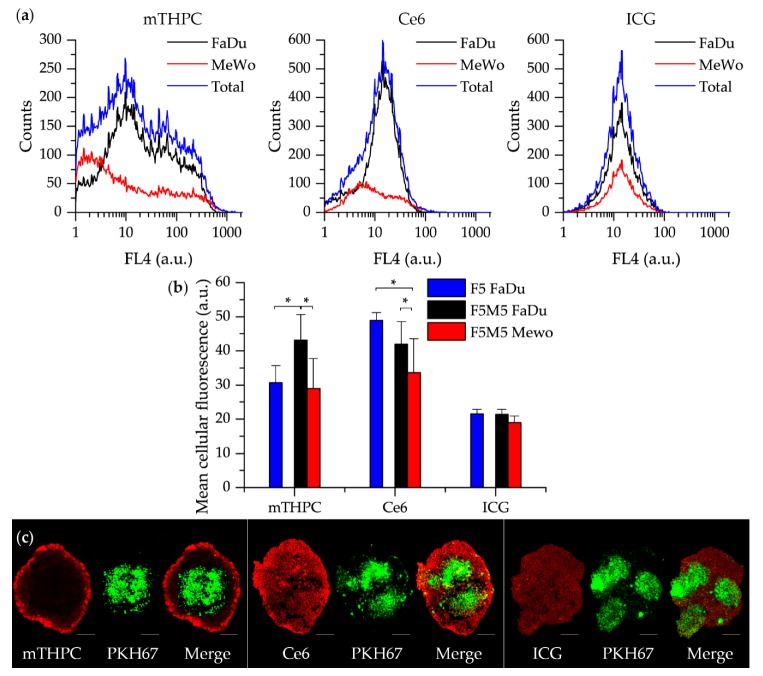
(**a**) PS distribution in FaDu and Mewo cells in co-culture FaDu:MeWo 5:5 spheroids at 24 h incubation. (**b**) The mean fluorescence intensity of spheroids treated with mTHPC, Ce6 and ICG at 24 h incubation. FaDu cells only from monoculture FaDu spheroids (blue column); FaDu cells from co-culture FaDu:MeWo 5:5 spheroids (black column); MeWo cells from co-culture FaDu:MeWo 5:5 spheroids (red color). (**c**) Typical fluorescence images of co-culture FaDu:MeWo 5:5 frozen-cuts, stained with PS (red) for 24 h. MeWo cells were pre-stained with PKH67 membrane dye (green). The concentration of dyes was 4.5 µM for mTHPC and Ce6 and 40 µM for ICG. *: statistically significant, *p* < 0.05. Scale bar—100 µm.

We further analyzed the distinct distribution of dyes in the populations of tumor FaDu cells and MeWo fibroblasts in FaDu spheroids. With this aim, MeWo cells were pre-stained with PKH67 membrane dye in order to identify MeWo fibroblasts in FaDu:MeWo 5:5 spheroids. It should be noted that the proportion of MeWo fibroblasts was 32 ± 2% to the total amount of cells in FaDu:MeWo 5:5 heterospheroids at day 5 post-seeding. Figure 7a displays the decomposition of total dye distribution profiles in two cell populations (FaDu tumor cells and MeWo fibroblasts). We observed a substantial accumulation of mTHPC in FaDu cell population, while in MeWo cells the PS fluorescence was significantly decreased, thus indicating mTHPC selective accumulation in tumor cells. Similar selective accumulation was also demonstrated for Ce6 (Figure 7a). On the contrary, ICG accumulates equally in both cell populations.

We further conducted a quantitative analysis of the accumulation of fluorescence dyes in each cell population of co-culture FaDu:MeWo 5:5 spheroids (Figure 7b). The uptake of mTHPC in FaDu cells in the presence of fibroblasts (co-culture FaDu:MeWo 5:5 spheroids) was significantly higher (*p* < 0.05) than that in FaDu homospheroids. At the same time, the accumulation of Ce6 in FaDu cells in the presence of fibroblasts (FaDu:MeWo 5:5 heterospheroids) was comparable with FaDu homospheroids. 

Among two cell populations in cocultured spheroids, mTHPC and Ce6 were accumulated significantly better in tumor FaDu cells (*p* < 0.05). ICG accumulation was not different between cell populations, nor spheroid types. To visualize the described distribution of dyes between various cell populations, representative fluorescence images of FaDu:MeWo 5:5 heterospheroids cryosections were introduced in Figure 7c.

#### 3.2.3. PDT Efficacy

**Figure 8:**
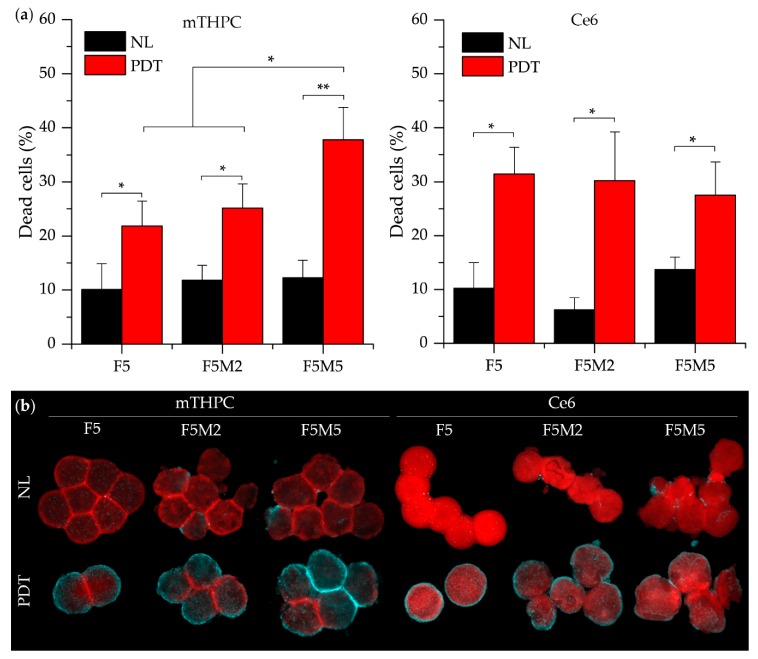
(**a**) The percentage of necrotic cells before and 6 h after PDT treatment (20 J/cm^2^) of FaDu monoculture and FaDu:MeWo (F5M2 and F5M5) co-culture spheroids with mTHPC and Ce6 (4.5 µM). (**b**) Typical fluorescence images of spheroids stained with PS (red) and annexin-V-FITC dye (cyan) as apoptotic marker without irradiation and 6 h after PDT (20 J/cm^2^). *: statistically significant, *p* < 0.05. Scale bar—500 µm.

mTHPC and Ce6 were further examined in terms of photo-induced damage. Phototoxicity was evaluated in spheroids after 24 h incubation with PSs and successive red-light irradiation (20 J/cm^2^) by means of flow cytometry and fluorescence microscopy (Figure 8). Toxicity in control, no light groups never exceeded 15%. PI-assessed mTHPC-photoinduced necrosis was significantly higher in co-culture FaDu:MeWo 5:5 spheroids compared with other experimental groups (*p* < 0.05) (Figure 8a). On the contrary, Ce6-mediated photoinduced cell death was not significantly different between spheroids with various stroma content and was about 30%. PDT-induced cytotoxicity was completed by fluorescence imaging of whole spheroids stained with FITC-Annexin V marker for apoptotic and necrotic cells (Figure 8b). The fraction of damaged cells (necrotic and apoptotic) is considerably increased in stroma-rich FaDu:MeWo 5:5 spheroids subjected to mTHPC-PDT compared to that of FaDu:MeWo 5:2 and FaDu spheroids. In contrast, the number of damaged cells in Ce6-PDT treated spheroids was visually less evident in FaDu:MeWo 5:5 heterospheroids compared to other spheroid types. 

## 4. Discussion

Tumor cells, grown as spheroids, more closely resemble in vivo solid tumors presenting physical and physiological barriers to drug action that monolayer cell cultures do not [37,38]. The physiological relevance of the model could be further increased by incorporating multiple cell types (e.g., fibroblasts [39,40,41,42], endothelial cells [39,43], macrophages [44,45,46]). Current advances have started to integrate the third cellular component like macrophages or endothelial cells in tumor/CAF heterospheroids. This tri-culture model better replicates the dynamic interaction between tumor, stromal and immune compartments [39,44,45,46]. In addition, while traditional three-dimensional tumor culture systems have relied on immortalized cell lines, patient-derived material is also used in the development of 3D tumor models [39,47,48]. Recently 3D co-culture tumor model based on stacked-layer cultures of HNSCC FaDu cells and patient-derived CAFs was configurated and assessed for radiation therapy [36]. However, as discussed recently, the culture of such cells in vitro is challenging due to difficulties in isolation and limited proliferative capacity [38]. In the present study, we developed stroma-rich 3D co-culture HNSCC spheroids consisting of FaDu (human pharynx squamous cell carcinoma) cells and MeWo granular fibroblasts, derived from human melanoma for testing of accumulation, distribution and PDT-mediated toxicity of photoactive drugs.

In the first part of the study, we characterized the FaDu/CAF heterospheroid model in terms of growth kinetics, cellular organization and expression of ECM markers. FaDu cells are strongly adhesive and due to this high adhesion of tumor cells upon spheroid growth, the density of cell packing increased while the size remained almost unchanged (Figure 1c). This observation is consistent with other studies, demonstrating that FaDu cells form spheroids with high packing density and uniform spherical shape [6,32,49,50,51,52,53]. Thus, the size of spheroids is mainly determined by the seeding concentration of FaDu cells. The addition of increasing seeding concentrations of MeWo cells only slightly changed the size of spheroids (Figure 1b). The size of heterospheroids was increased only by 3% and 10% for F5M2 and F5M5 compared to FaDu homospheroids at 5 day post-seeding because CAFs exhibits even higher adhesion than tumor cells, forming large clusters inside of heterospheroids (Figure 1c). Summarizing the literature data on this issue [39,41,54], the growth kinetics of spheroids strongly depend on the cell lines and the spheroid formation procedure.

According to immunochemical analysis, the addition of fibroblasts increased the total number of cells in spheroid (Figure 2). Indeed, the necrotic core appeared more rapidly in heterospheroids compared with homospheroids. Since the size of heterospheroids changed only slightly, while the number of cells was increased, one can suppose that cellular density in FaDu/MeWo spheroids is higher than that in FaDu homospheroids. Hence, the gradients of nutrients could be established much faster in heterospheroids compared to homospheroids, resulting in faster formation of gradient of proliferation and necrotic core in co-culture spheroids. It is worth noting that PKH67 staining of MeWo becomes less contrast over time due to the proliferation of MeWo cells and redistribution of PKH67 to tumor cells. In HNSCC, CAFs frequently have this myofibroblastic phenotype (α-SMA positive) [35,55] and are associated with dense collagen deposition and stromal desmoplasia [56]. Detailed investigation of stroma markers in spheroids at day 5 post-seeding demonstrated the lack of deposition of ECM components in FaDu monospheroids, while co-culture spheroids exhibited a strong expression of collagen and fibronectin as well as that of CAF biomarkers α-SMA and vimentin. It should be noted that vimentin staining strongly correlates with PKH67 fluorescence staining of MeWo cells. According to the flow cytometry analysis, the ratio between MeWo fibroblasts and tumor FaDu cells was 1:2 on the 5th day of coculturing. However, the presence of even 30% stromal cells resulted in the expression of ECM components such as fibronectin and collagen across the whole volume of heterospheroids. 

For drug screening, we selected spheroids on day 5 post-seeding since a good balance between cell viability and stroma-rich environment was achieved at that time. Drug physiochemical properties strongly affect its distribution in tissues, thus we selected photoactive drugs with different physicochemical characteristics to evaluate the effect of stroma content on the drug behavior in tumor spheroids. We compared the distribution and localization of three above mentioned fluorophores in FaDu homospheroids and stroma-rich FaDu:MeWo heterospheroids. In addition, mTHPC and Ce6 were tested for their photo-induced antitumor efficiency in homo- and heterospheroids. It should be noted that photoactive drugs used in this study have different photophysical and spectral characteristics, exhibiting red (mTHPC, Ce6) and near infra-red (ICG) fluorescence. Thus, quantitative comparison between the dyes should be avoided. Below we summarized the main effects of stroma content on the drug behavior in tumor tissue.

- mTHPC. mTHPC is a highly hydrophobic and lipophilic molecule, characterized by limited penetration in tumor spheroids [57,58,59,60]. The addition of stroma had a positive outcome on the distribution of mTHPC, increasing mTHPC overall incorporation in MCTSs with a preferential accumulation in FaDu tumor cells. mTHPC releases very slowly from the cells, thus its rapid propagation across the spheroid is unlikely. This molecule usually distributes by means of serum lipoproteins in interstitial space, therefore, the enhanced penetration in heterospheroids could be related to the expansion of interstitial space in stroma-rich spheroids by stromal components. An interesting observation is that mTHPC exhibits selectivity towards FaDu tumor cells vs. CAFs. Finally, a significantly better PDT response in stroma-rich FaDu:MeWo 5:5 spheroids was obtained compared with FaDu homospheroids.
- Ce6. Contrary to mTHPC, Ce6 is more hydrophilic and penetrates more easily into spheroids [61,62,63]. Ce6 penetration in homospheroids requires 24 h, while in heterospheroids this process is accelerated and complete distribution is achieved at already 6 h. Similar to mTHPC, Ce6 possesses selectivity against FaDu tumor cells, however the overall effect of the presence of stromal components on Ce6 accumulation is negative (Ce6 accumulation in heterospheroids is lower than that in homospheroids). Thus, stroma components seem to be an additional barrier for Ce6 penetration in tumor tissue [64]. As a result, PDT efficiency of Ce6 in stroma-rich heterospheroids is not significantly different from that in homospheroids.
- ICG. An anionic, amphiphilic & water-soluble fluorophore ICG quickly distributes across any type of spheroids independently on the presence of stroma components. In contrast to PSs such as mTHPC and Ce6, ICG has no selectivity between CAFs and tumor cells.

## 5. Conclusions

We have successfully developed a novel 3D stroma-rich in vitro model of HNSCC, using cancer-associated MeWo skin fibroblasts and FaDu pharynx squamous cell carcinoma. We confirmed the expression of stromal components in heterospheroids and successfully applied this co-culture tumor model for the screening of photoactive drugs. We clearly demonstrated the potential of developed stroma-rich spheroids for studying the impact of tumor-stroma interactions on the antitumor effects of photoactive drugs.

Stromal components especially CAFs are the abundant constituents in HNSCC, exerting in some cases a physical barrier for anti-cancer therapeutics penetration and thus limiting their antitumor activity. Indeed, Ce6 accumulates less in co-culture spheroids compared with FaDu homospheroids, while mTHPC displays quite different behavior. mTHPC had a better PDT efficiency in stroma-rich tumor tissue due to the better accumulation, thus confirming a pivotal interest in using this molecule in the management of HNSCCs. As for ICG, its equal distribution in both stroma-rich and monoculture spheroids confirms its efficacy irrespective of the stroma content of HNSCC.

Overall, we can conclude that the presence of stroma influences photoactive drugs in different ways. To our knowledge, no analogous MCTS model combining HNSCC cells and stroma components has been previously constructed. As such, the developed stroma-rich spheroids enlarge the arsenal of in vitro pre-clinical models for high-throughput screening and could better predict in vivo distribution of drugs with various stroma content in tumor tissue. Our ongoing study assesses the behavior of photoactive nanoparticles in stroma-rich HNSCC spheroids in order to delineate the role of stromal components on the nanoparticle-based delivery of anti-cancer drugs.
